# A circularly polarized graphene based wideband 1 × 2 array antenna for terahertz spectrum applications

**DOI:** 10.1016/j.heliyon.2024.e37575

**Published:** 2024-09-07

**Authors:** Abdelaaziz El Ansari, Sudipta Das, Tanvir Islam, Varakumari Samudrala, Naglaa F. Soliman, Abeer D. Algarni, Najiba El Amrani El Idrissi

**Affiliations:** a Signal, System and Component Laboratory, Sidi Mohamed Ben Abdellah University – FST, Fez, 30000, Morocco; b Department of Electronics & Communication Engineering, IMPS College of Engineering and Technology, Malda, 732103, WB, India; c Department of Electrical and Computer Engineering, University of Houston, Houston, 77204, TX, USA; d Department of Electronics and Communication Engineering, NRI Institute of Technology, Agiripalli, Vijayawada, Andhra Pradesh, 521212, India; e Department of Information Technology, College of Computer and Information Sciences, Princess Nourah bint Abdulrahman University, P.O. Box 84428, Riyadh 11671, Saudi Arabia

**Keywords:** Array antenna, Circular polarization, Flower slots, Patch antenna, THz band, Terahertz antenna

## Abstract

A graphene-based 1 × 2 array antenna with circular polarization for terahertz applications is prescribed in this article. Initially, a novel concept of a folded quarter wave impedance transformer is utilized in the design process of a single element for minimizing the overall antenna size. The opposite corners of the patches have been truncated and structural modifications are performed with the insertion of four flower-shaped slots along with an additional circular slot for achieving a much-improved reflection coefficient and better impedance bandwidth. It also shows a much wider 3 dB axial ratio bandwidth, confirming circular polarization due to the suggested modifications in its geometry. Then, an array antenna has been formed to provide better gain. The configured patches are fed by a magic-T power divider to attain the required impedance matching. The results of the CP antenna array have been analyzed using the HFSS and CST simulators. The propounded 1 × 2 array antenna shows circular polarization with a 3 dB AR bandwidth of 205 GHz (2.345–2.55 THz) and wide spectral coverage of 210 GHz (2.345 − 2.555 THz) along with a maximum gain of 8.65 dB and 99.8 % radiation efficiency with a total size of 53.5 × 102 × 1.56 μm^3^. It could be utilized for high-speed data transmission, material characterization, terahertz spectroscopy, terahertz imaging, etc. applications.

## 1 Introduction

The terahertz (THz) band has tremendous potential applications for numerous fields including both outdoor and indoor communications within the spectrum of 0.1–10 THz. Moreover, it promises significant applications in many areas specifically in Manufacturing, agriculture, materials characterization, spectroscopic imaging, and security [1]. Due to the availability of unallocated enormous bandwidth, the terahertz band supports significant high-speed communication by implementing effective terahertz antennas [2,3], which is impossible to reach in the microwave band that is allocated and congested with specified applications. However, there are several factors including high atmospheric attenuation and path loss which meanwhile limit the use of the entire THz spectrum effectively. In this context, high gain and highly directive antennas are supremely required to overcome these problems and to permit efficient transmission of data over long distances.

Over the last few decades, the dominance of printed circuit technology has been evident and as a result of this, microstrip patch antennas (MPAs) are highly preferred for the successful establishment of wireless communication systems from Radio Frequency (RF) to THz frequency bands. Further, microstrip patch antenna (MPA) has various advantages and also suffers from several drawbacks. Therefore, many scientists and researchers have reported several techniques to overcome the deficiencies (limited bandwidth, linear polarization, low directivity and gain, etc.) of the MPA, such as modified patch and ground structures [[4], [5], [6]], multilayer substrate [7], Substrate integrated waveguide structures [8], metamaterials [9,10], electromagnetic band gap (EBG) [11,12], and Micro-Electro-Mechanical Systems (MEMS)-based antenna structures [13], etc. The photoconductive antennas are a pivotal part in the generation and reception of terahertz (THz) radiations; however, due to critical impedance matching issues, they are associated with drawbacks of low output power. To resolve this issue, microstrip patch antennas are alternatively preferred to support applications in different spectrums of terahertz frequency regimes. In the literature, researchers have adopted and explored many new design methodologies for designing various geometries of printed terahertz antennas which include array antenna configurations [14,15], graphene-based simple patch [16], tree-shaped patch [17] photonic crystal antenna [[18], [19], [20]], proximity coupled monopole antenna [21], square ribbon patch with the defected ground and Photonic band-gap (PBG) substrate [22], modified patch with slotted ground [23], Metasurface inspired antenna [24], curved slit loaded patch with CPW feeding [25], metamaterial antenna [26], antenna with PBG structure over SiO2 substrate [27], octagonal patch on SiO2 substrate [28], 2 × 2 Multiple-Input Multiple-Output (MIMO) [29], semi-circular moon shaped radiator [30], fractal antenna [31], antenna with semi-circular slot [32], fractal MIMO antenna [33], rhombus-shaped patch radiator [34], novel coronavirus shaped patch [35].

Although these techniques have successfully managed to design terahertz antennas with acceptable characteristics and parameters, these structures are restricted against polarization i.e., the antennas are arbitrarily orientated and linearly polarized. Nowadays, the most demandable antennas are the antennas having a circular polarization (CP) due to their ability to transmit electromagnetic waves in any direction [[36], [37], [38], [39], [40], [41], [42], [43]]. The referred [[36], [37], [38], [39], [40], [41], [42], [43]] design methodologies are suggested to achieve circular polarization for microwave and mm-wave application bands. In a likewise manner, there are high demands for circularly polarized THz antennas for supporting numerous applications at different terahertz frequency spectrums as recommended and reported in Refs. [[44], [45], [46], [47], [48]]. It includes an S-shaped and Z-shaped slots-loaded CP antenna structure [44], a rectangular dielectric resonator CP antenna (DRA) [45], an E-shaped transparent antenna [46], an optical transparent antenna perturbed with E and I-shaped slots [47], modified circular antenna with plexiglass superstrate material [48], leaf-shaped graphene antenna [49], dielectric resonator antenna with crossed slot [50], etc.

Based on the literature studies, there is a necessity to propose a circularly polarized terahertz antenna with superior performance parameters. In this context, the authors of this current article aimed to design an array antenna to offer wide bandwidth, circular polarization (CP) characteristics, high gain, and radiation efficiency by maintaining its compact size. The proposed array configuration has been adopted to achieve CP characteristics using a single feed technique. The corner truncated optimized patches are modified by adding flower slots and a circular slot to get an ideal circular polarization with enhanced performance. Finally, these two patch elements are fed together by a T magic power divider which increases the impedance bandwidth, 3 dB axial ratio bandwidth, radiation efficiency, and gain. The prescribed array antenna is dedicated to function for wireless communications in the Terahertz (THz) band, especially at around 2.45 THz. Consequently, it can be utilized for high-speed data transmission, material characterization, terahertz spectroscopy, and terahertz medical imaging applications.

The major highlights of the presented terahertz array antenna are summarized:

i. **Miniaturized size:** The designed single feed 1 × 2 array antenna holds a tiny area of only 53.5× 102 μm^2^.
ii. **Design Methodology:** Folded feed line, corner truncation, loading of flower and circular shaped slots, array antenna formation, and use of magic-T power divider.
iii. **Wideband operation:** It is characterized by a wide operating bandwidth of 210 GHz, spanning from 2.345 THz to 2.555 THz at a reference of 10 dB return loss level.
iv. **Wide Axial Ratio bandwidth:** It shows a wide 3 dB ARBW of 205 GHz [2.345 − 2.55 THz].
v. **Circular Polarization:** The designed array shows circular polarization characteristics covering a large terahertz frequency spectrum.
vi. **High Radiation Performance parameters:** It offers a high gain of 8.65 dB and radiation efficiency reaches a maximum of 99.8 % at 2.45 THz.
vii. **Applications:** The suggested antenna could be utilized for high-speed data transmission, material characterization, terahertz spectroscopy, terahertz imaging, etc. applications.

After this introduction part, the discussion on graphene material is carried out in section 2. The design methodology for the proposed single radiating element is discussed in section 3. Section 4 demonstrates the design and simulation results of the single-fed circularly polarized array antenna. After that, the validation of the design and results with Computer Simulation Technology (CST) software is presented in section 5. Next, in section 6, an extensive performance comparison analysis with state-of-the-art is analyzed. Finally, section 7 concludes the article concisely.

## 2 Discussion on graphene material

**Fig. 1:**
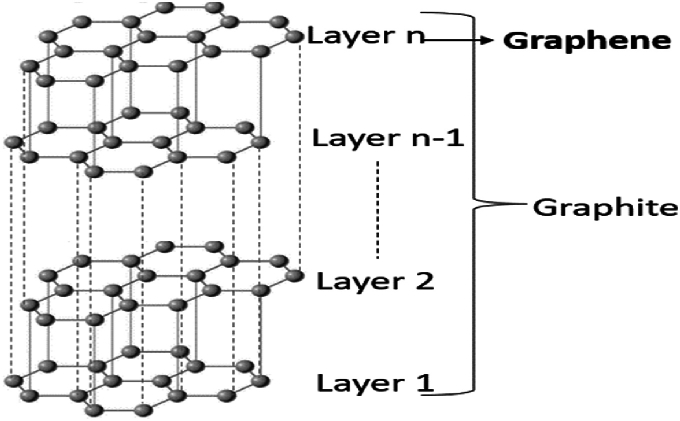
Graphite and graphene carbon structure.

Graphite is the most well-known allotropes of carbon due to its excellent conductivity. In graphite, the graphene sheets are organized as flat hexagonal layers, which consist of an arrangement of carbon atoms (Fig. 1). These layers are clutched with the help of the Van Der Waals forces when they pile on top of one another. Graphene is a widely popular 2D material due to its remarkable mechanical and electrical qualities. Scientists have been paying prime focus to graphene to unlock its excellent potential for the modeling of next-generation devices, to be utilized in the terahertz spectrum. It is a highly versatile material with outstanding conductivity, mechanical strength, and flexibility, and is suitable for numerous technological advancements. Its critical parameters (conductivity, chemical potential, relaxation time, thickness, mechanical strength, flexibility, and optical properties) make it promising for a wide variety of applications, which include photonics, electronics, etc. [16]. The graphene-based antennas are useful for many applications in the THz frequency spectrum. Graphene is preferred as a conductive material for high-frequency (THz) applications with low energy consumption. Due to its unique structure, it offers high charge mobilities, allowing fast-moving electricity compared to other metals. This distinctive property creates a special form of EM radiation in the THz frequency band. The essential parameters used in describing the properties of graphene material are summarized as follows:

i. **Thickness:** The thickness of graphene is indicated by the single-layer thickness of carbon atoms. The thickness approximately equals to 0.34 nm (nm).
ii. **Relaxation time:** It is the average time between scattering events that an electron experiences as it moves through the graphene lattice. its value can range from picoseconds (ps) to nanoseconds (ns).
iii. **Chemical Potential:** Its chemical potential refers to the Fermi level relative to the Dirac point, which can be adjusted by doping or applying a gate voltage. It can range from −1 eV to 1 eV, but it is often controlled within a smaller range around the Dirac point (0 eV) for many applications.
iv. **Electrical Conductivity:** It is featured with high electrical conductivity and electron mobility. It can conduct electricity better than many other materials due to its unique electronic structure, which allows electrons to move through it with minimal scattering.
v. **Thermal Conductivity:** Graphene has excellent thermal conductivity, which can exceed 3000 Wm^−1^K^−1^. This makes it highly efficient for heat dissipation in electronic devices.
vi. **Mechanical Strength:** Graphene is extremely strong. Despite being only one atom thick, it is incredibly robust and can withstand significant mechanical stress.
vii. **Flexibility:** Graphene is highly flexible and can be bent, twisted, and stretched without breaking. These qualities make it suitable for applications that require both flexibility and strength.
viii. **Optical Properties:** It is nearly transparent and can absorb up to 2.3 % of visible light. This property, combined with its electrical conductivity, registered it as a dream fit for use in conductive films with transparency and other optoelectronic applications.
ix. **Surface Area:** Graphene has a high surface area (2630 m^2^/g), which makes it advantageous for applications in sensors, energy storage, and catalysis.

## 3 Basic radiating element design methodology

**Fig. 2:**
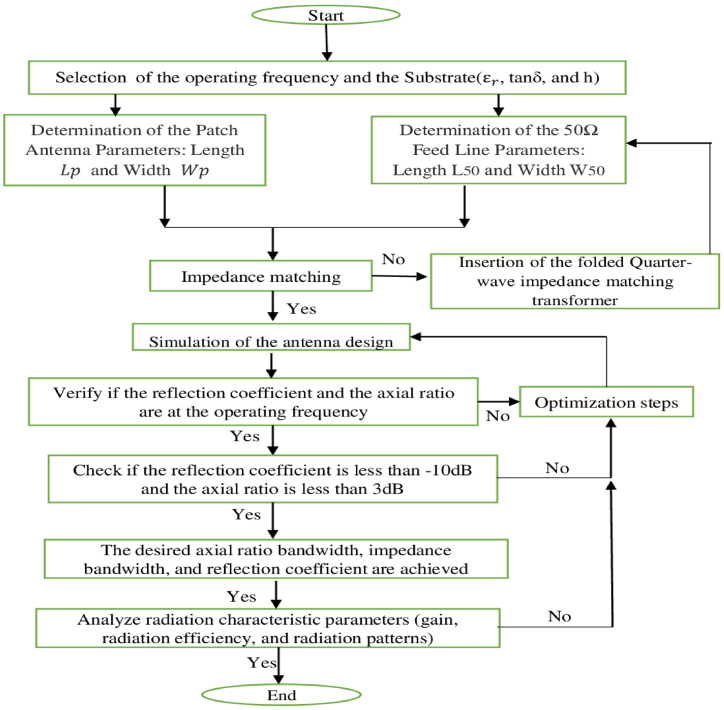
Antenna design methodology flowchart.

The initial radiating element is a rectangular-shaped patch antenna (RSPA). Its dimensions can be determined through equations that exist in Refs. [[51], [52], [53], [54]]. The methodology used to obtain the proposed circularly polarized basic antenna is presented in the form of a design flowchart [55], shown in Fig. 2. The initiated basic element is fed by a folded impedance matching transformer to get a minimized antenna size. The intended shape of the single-element antenna has been reached after following seven optimization steps through the incorporation of truncated corners, flower-shaped slots, and a circular slot. Seven design steps are used to obtain a resonating frequency at 2.45 THz with an improved return loss and axial ratio. This proposed design is conducted on Rogers' substrate (RT/duroid 5880) with a total dimension of 50 × 51× 1.56 μm^3^. The Rogers’ substrate is highly reliable and it has low electrical losses, low moisture absorption, and stable dielectric constant over a wide range of frequencies. Graphene is employed as a conducting material for designing both the ground plane and the radiating element due to its magnificent electrical, thermal, and mechanical properties. The intended design stages to obtain the propounded antenna structure are demonstrated in the succeeding sections.

### 3.1 Effect of truncated corners

**Fig. 3:**
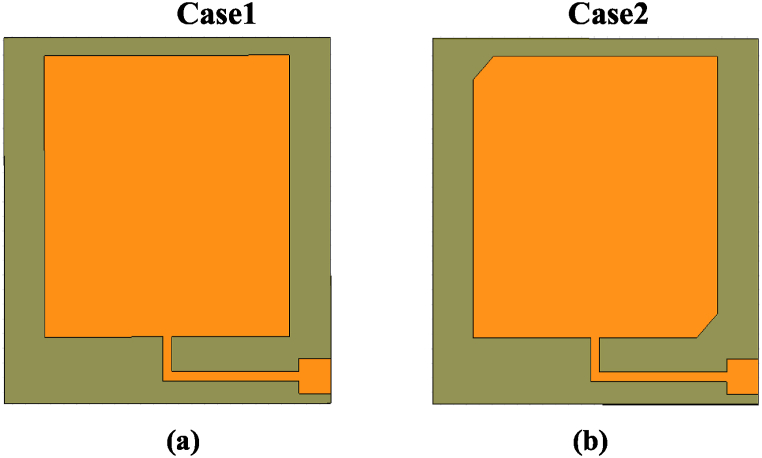
(a) The basic element fed by a folded quarter wave impedance transformer. (b) The basic element with two truncated corners.

**Fig. 4:**
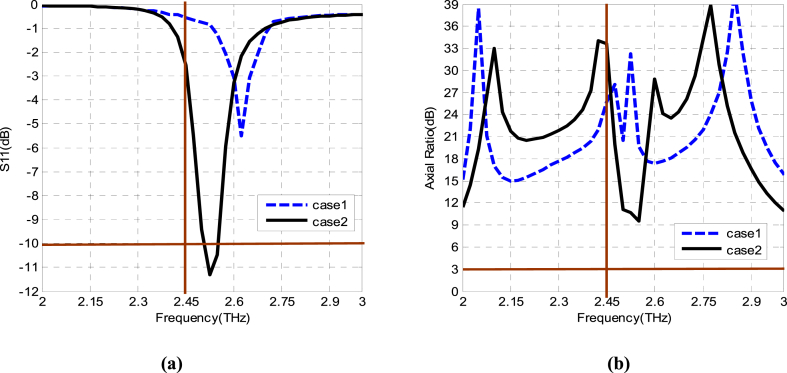
Simulation results of case 1 versus case 2. (a): S_11_ (b): The axial ratio.

Initially, the basic radiating element is depicted in Fig. 3 (a), and its geometrical dimensions are included in Table 1. First of all, in Case 1, the proper impedance matching has been assured by a quarter wave impedance transformer in a folded shape to achieve minimum antenna size as displayed in Fig. 3(a). Its reflection coefficient is displayed in Fig. 4 (a) which signifies a resonance peak at 2.62 THz with an S_11_ of about − 5.5 dB. Moreover, its axial ratio variations are depicted in Fig. 4 (b), according to the curve, it has values tending to the maximum limit (much above 3 dB) which confirms that the design in Case 1 has a linear polarization. To make the basic element a circularly polarized, 90° phase differed two orthogonal resonant modes of equal magnitudes should be generated. In the next steps, some modifications are introduced in design steps that can help to generate these orthogonal modes.

**Table 1:** Optimal geometrical parameters of the basic antenna.

Parameters	L_S_	W_S_	L_P_	W_P_	F_L1_	F_L2_	F_L3_	W_F1_	W_F2_	D	d
Value (μm)	50	51	38.42	38.22	10	21.43	5	1.35	4.84	10.4	4.5

The first stage for improving the performance of the structure presented in Case 1 is to truncate two corners at two opposite sides as shown in Case 2 of Fig. 3 (b). These two truncated corners perturb the patch along its diagonal axes and make an asymmetric structure. Consequently, it leads the basic element near the circular polarization and offers significant impedance matching and shifting of resonance to a lower value. Fig. 4(a and b) shows its reflection coefficient S_11_ versus frequency as well as its axial ratio versus frequency for Case 2. The reflection coefficient is improved to − 11 dB at 2.52 THz and also, the axial ratio is down to 9 dB at 2.54 THz.

### 3.2 Effect of flower slots

**Fig. 5:**
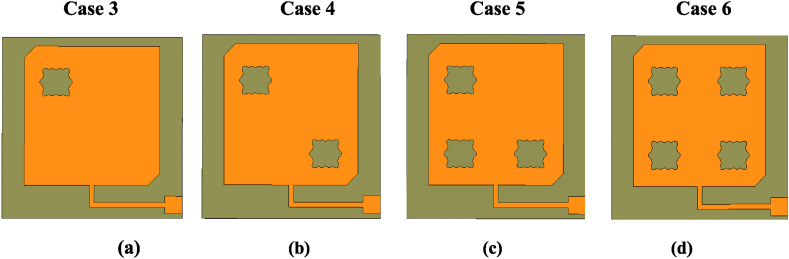
Flower-shaped slots loaded design cases (a) one flower slot (b) two (c) three (d) four flower slots.

**Fig. 6:**
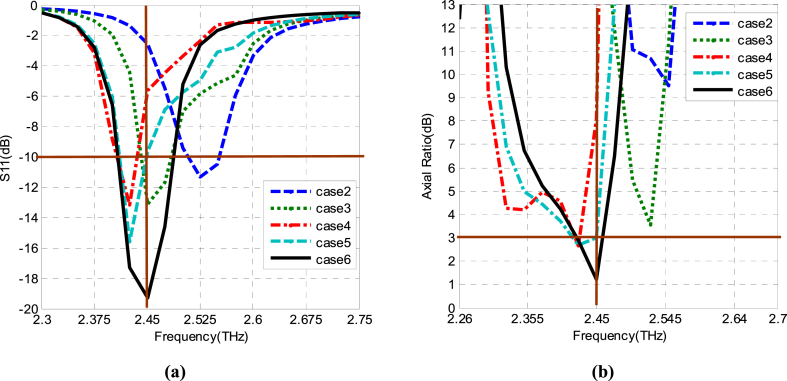
Simulation results of Case 2, 3, 4, 5, and 6. (a): S_11_ (b): Axial ratio.

In Case 2, the values in terms of S_11_ (=− 11 dB) and AR (=9 dB) aren't enough to obtain a wideband antenna with good circularly polarized waves. In the third step (Case 3), the objective is to resonate the basic element at the desired frequency (2.45 THz) with improved performance. The first flower-shaped slot is introduced within the patch as observed in Case 3 [Fig. 5]. As seen, the resonant frequency is further moved to a lower value, but the minimum value of the AR is not reached to 3 dB at the desired frequency of 2.45 THz [see Fig. 6(b)]. To achieve it, the next step is executed in which a second flower slot is added in the opposite quarter (see Fig. 5, Case 4). As shown in Case 4 of Fig. 6, the resonance is further lowered and decreased to 3 dB value as per the AR vs frequency plot. After that, a 3rd flower slot is added in the 3rd quarter as depicted in Case 5 of Fig. 5. Now, concerning both S_11_ and AR, the resonant frequency is achieved at the same value but not at the desired value of 2.45 THz as shown in Fig. 6 [Case 5]. In this context, the 4th flower slot is added (see Fig. 5, Case 6). In this case, the resonant frequency at 2.45 THz is obtained for both AR and S_11_ plots. The antenna shows S_11_ = − 19 dB, and AR = 1.3 dB at 2.45 THz with an improved − 10 dB IBW and 3 dB ARBW as compared to the preceding design cases.

### 3.3 Effect of circular slot

**Fig. 7:**
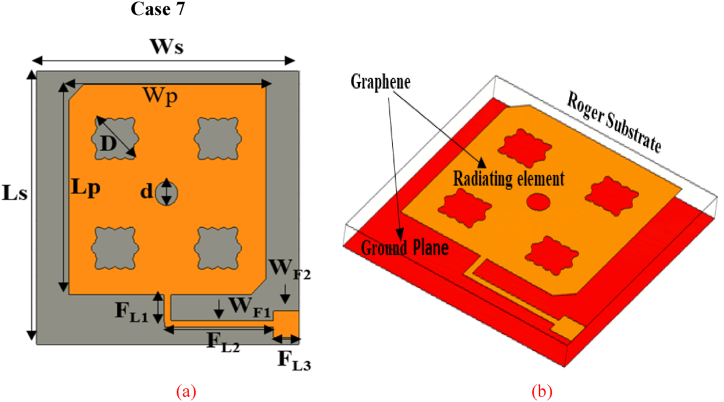
Proposed flower-shaped and circular slots-based CP antenna views (a) 2D (b) 3D.

In addition to the previous six improvement steps, in this section, we present the last improvement step designated as Case 7. A circular slot is introduced in this design stage, as shown in Fig. 7. The optimal radius of this circular slot is obtained after the parametric sweep. This slot is used for the enhancement of bandwidth as per both reflection coefficient S_11_ and axial ratio, as well as for decreasing their minimum values. Also, the gain and the radiation efficiency in this case are slightly enhanced due to the presence of an optimized circular slot. The geometrical attributes of the optimized basic antenna are summarized in Table 1.

**Fig. 8:**
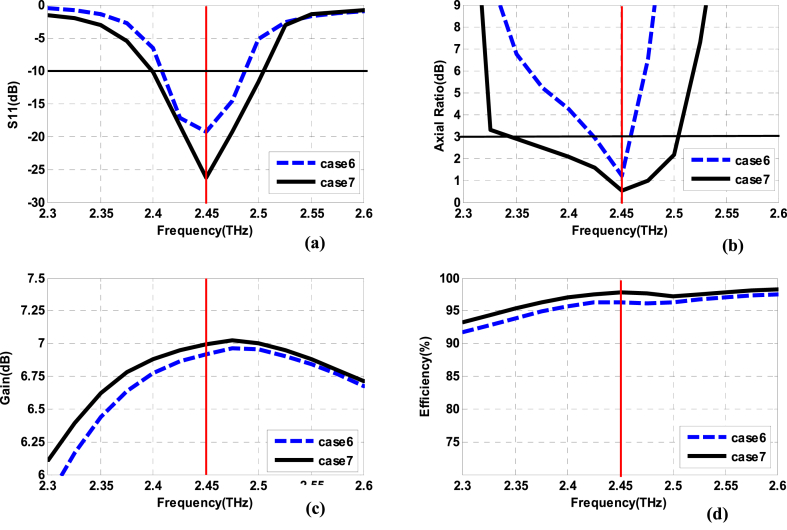
Simulation results of the proposed single-fed circular polarized basic element (a): reflection coefficient bandwidth. (b): Axial ratio bandwidth. (c): Gain versus frequency. (d): Radiation efficiency versus frequency.

The results of the simulation study for the basic element (comparison of Case 7 against Case 6) are exhibited in Fig. 8. As shown, the performance parameters achieved in Case 7 are improved compared to Case 6. The reflection coefficient is improved to − 24 dB with a little bit of increment in impedance bandwidth at − 10 dB reference level. However, prominently, the 3 dB ARBW is enhanced from 35 GHz to 165 GHz along with a little bit of improvements in the peak gain and radiation efficiency as well.

**Fig. 9:**
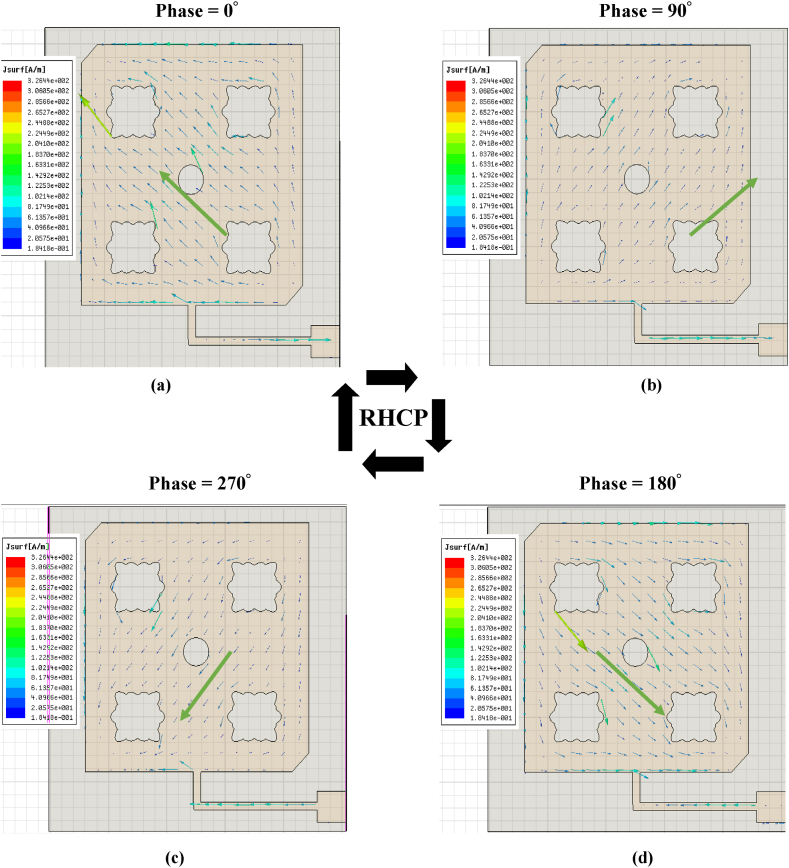
Surface current distribution 2.45 THz with the phase (a) 0° (b) 90° (c) 270° (d) 180°.

The surface current distribution of the optimized basic element with phases 0°, 90°, 180° and 270° at 2.45 THz are shown in Fig. 9. The current vector tip direction changes clockwise which means that the basic element reveals a right-hand circular polarization (RHCP) characteristic. These flower slots and the circular slot helped to perturb the distribution of surface current in the patch. Therefore, two orthogonal degenerate modes of resonance frequency with equal amplitudes are excited.

### 3.4 Parametric variations analysis

**Fig. 10:**
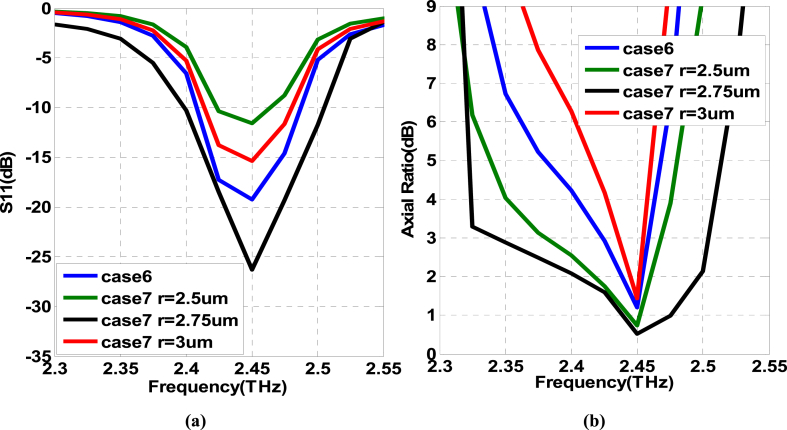
Variations in operating bandwidth as a function of circular slot radius ‘r’ (a) S_11_ (dB) (b) Axial Ratio (dB).

The outcome of variations in the radius of the incorporated circular slot has been examined through a simulation tool. It can be visualized that the addition of a circular slot within the patch structure is a very crucial design step. The variations in its radius (r) control the bandwidth of the antenna very significantly. The variations in S_11_ (dB) and Axial Ratio (dB) as a result of changes in ‘r’ are demonstrated in Fig. 10(a and b), respectively. It is noticeable that maximum IBW and 3-dB ARBW are achieved for the proposed dimension of r = 2.75 μm as indicated by the black solid line. The bandwidth performance is getting worse with further increases or decreases in its dimensions. Hence, r = 2.75 μm is considered to be the optimal circular slot radius during the design process.

## 4 Proposed 1 × 2 array antenna

**Fig. 11:**
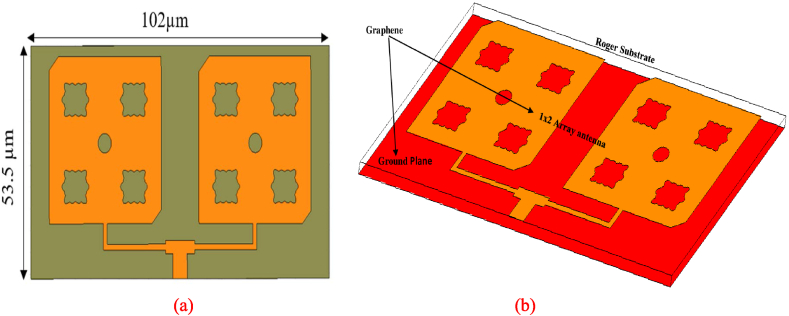
Proposed single fed circular polarized 1 × 2 array antenna views (a) 2D (b) 3D.

The design and simulation outcomes of the proposed graphene-based single-fed circularly polarized 1 × 2 array antenna is discussed in this section. For the excitation of the array, a simple T-power divider is employed for feeding the patch elements to get a well-adopted impedance matching [Fig. 11]. The performance of this array is analyzed to justify improvements in performance parameters (gain, impedance bandwidth, radiation efficiency, and axial ratio bandwidth). The intended array design is executed on the Rogers’ substrate (RT/duroid 5880) with an overall size of 53.5 × 102× 1.56 μm^3^.

**Fig. 12:**
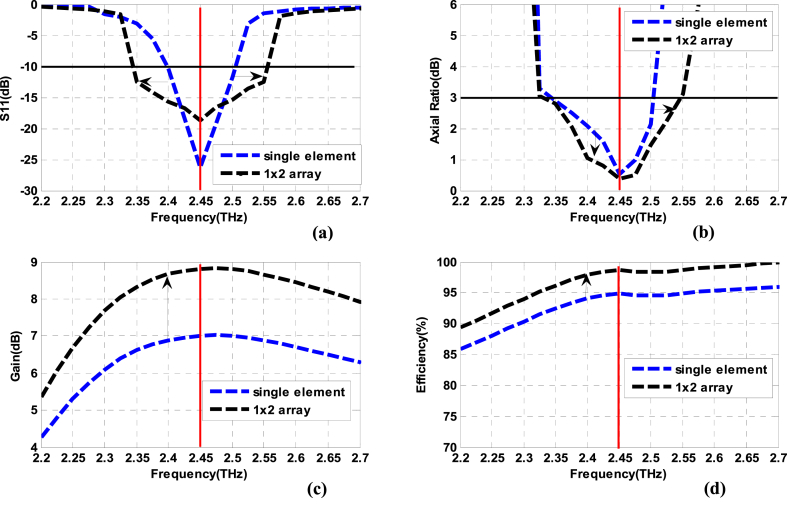
Simulation results of the single-fed circularly polarized 1 × 2 array antenna versus CP single element. (a) S_11_ (b) Axial ratio bandwidth (c) Gain, and (d) Radiation efficiency.

**Fig. 13:**
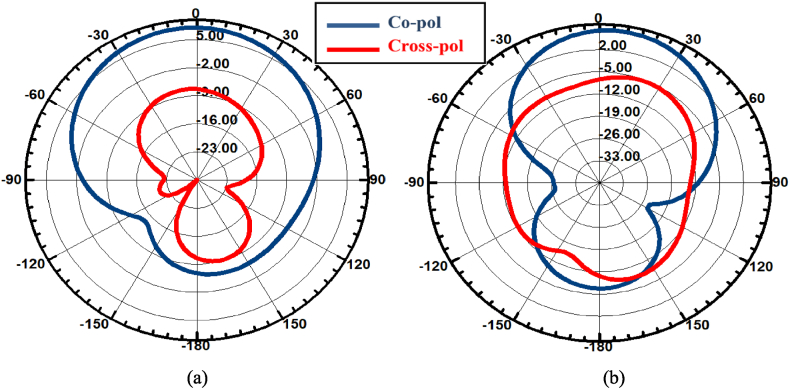
Radiation patterns representing Co-pol and Cross-pol at 2.45 THz in (a) E plane (b) H-plane.

The simulation studies have been performed by HFSS EM solver. Fig. 12 (a) shows the reflection coefficient characteristics which shows the bandwidth of the array antenna is well improved and equals 210 GHz (from 2.345 THz to 2.555 THz). The axial ratio plot is shown in Fig. 12 (b). The 3 dB ARBW is also significantly enhanced and it equals 205 GHz (2.345 − 2.55 THz). The gain versus frequency is illustrated in Fig. 12 (c), according to this figure, it is noticed that the peak gain is increased to 8.65 dB at 2.45 THz. Fig. 12 (d) presents the radiation efficiency; it is increased to 99.97 % at 2.45 THz. The radiation patterns are presented indicating both co and cross-pol components. The patterns are projected in Fig. 13(a and b). The patterns are obtained in the desired shape and the cross-pol levels are adequately down in comparison to co-pol in both planes.

**Fig. 14:**
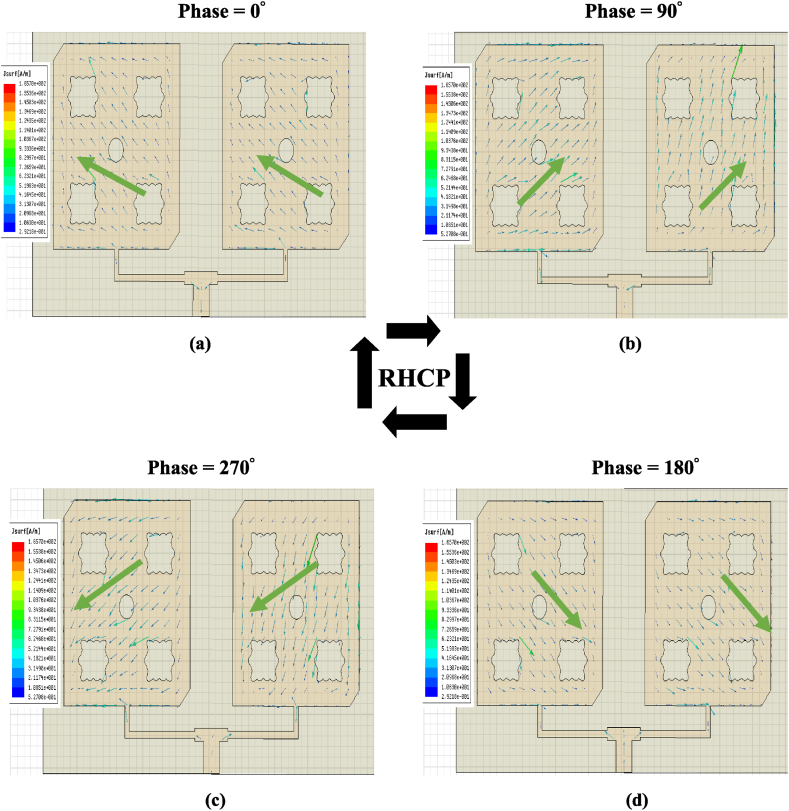
Distributed surface currents at 2.45 THz with (a) 0° (b) 90° (c) 270° (d) 180° phases.

In addition, the circular polarization (CP) is evaluated by demonstrating surface current distribution at 2.45 THz as shown in Fig. 14. The CP is obtained by the excitation of two orthogonal modes having a phase shift of 90°. This is accomplished by the single feed, corners truncated, flower slots, and circular slots inserted on the patch element. In surface current distributions, figures with 0°, 90°, 180°, and 270° phases are plotted. Thus, it is clear that the direction of the vector current tips rotates in a clockwise direction, which proves the RHCP (Right Hand Circular Polarization) in the boresight direction.

## 5 Validation of design and results with computer simulation technology (CST) software

As per the current scenario, fabrication and measurement of THz antennas are challenging because of tiny dimensions and non-available practical resources. The various processes like PCB etching [56], nano-lithography [57], and micro-machining [58] can be implied to execute the practical prototyping of the THz antenna. The PCB etching process needs a high-precision etching module to execute the design with maximum accuracy. The nano-lithography and micro-machining techniques are employed to design or etch the microscopic level structures.

**Fig. 15:**
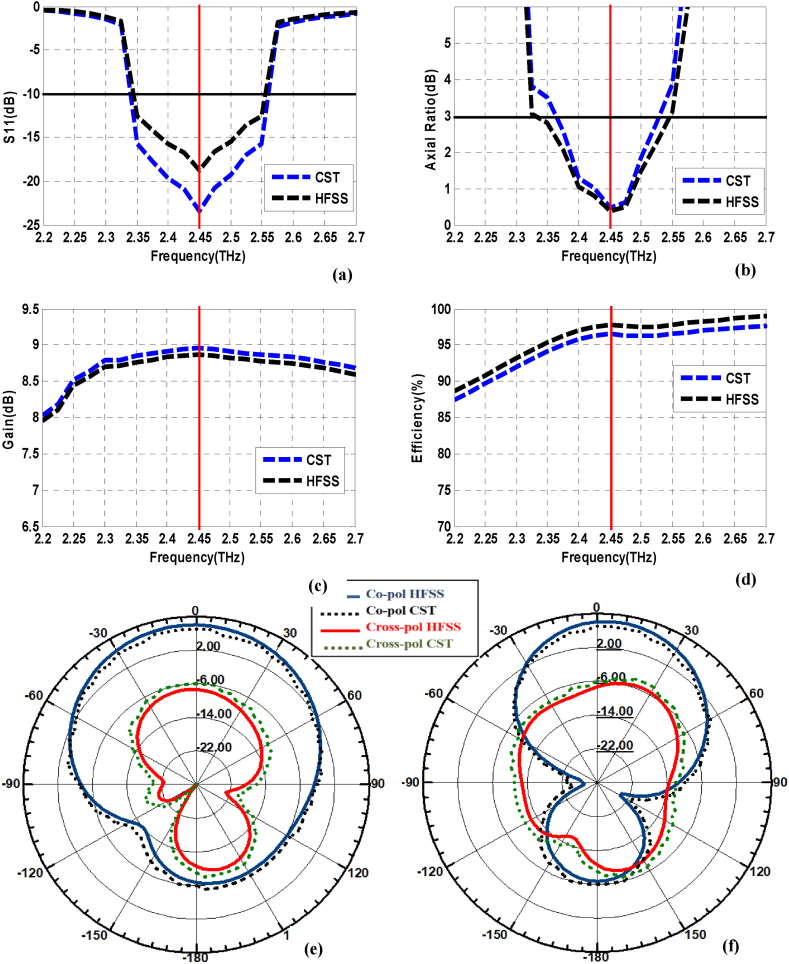
Comparison of HFSS and CST Simulation results for 1 × 2 array antenna. (a) S_11_ (b) Axial ratio bandwidth (c) Gain (d) Radiation efficiency, (e) E plane, and (f) H-plane radiation patterns.

Due to the unavailability of resources for prototype testing and validation, we have carried out the design and analysis using two different 3D EM simulators (HFSS and CST). Both 3D simulators are used for electromagnetic problems. These tools are widely used for designing antennas, microwave circuits, RF components, and other high-frequency devices. The design and simulation analysis of the array antenna is executed by utilizing an HFSS-EM solver. The results obtained from HFSS software are validated by analyzing the results of the prescribed array antenna using the CST tool. Fig. 15 clarifies good agreement between the outcomes obtained from both EM solvers, which confirms the validity of the designed array antenna for terahertz applications.

## 6 Performance analysis with reported terahertz antennas and discussion

The performance of the suggested circularly polarized high gain, highly efficient terahertz array antenna is compared with a few previously referred terahertz antennas [[14], [15], [16], [17], [18], [19], [20], [21], [22], [23], [24], [25], [26], [27], [28], [29], [30], [31], [32], [33], [34], [35],[44], [45], [46], [47], [48], [49]] in terms of antenna dimensions, operating band, impedance bandwidth, ARBW, gain, and polarization. The summary of performance comparison analysis is presented in Table 2. As compared with other existing linearly polarized (LP) THz antenna designs [[14], [15], [16], [17], [18], [19], [20], [21], [22], [23], [24], [25], [26], [27], [28], [29], [30], [31], [32], [33], [34], [35]], the proposed array antenna is considerably miniaturized. The proposed array antenna provides the highest gain as compared to all other references except ref. [30,32]. However, it offers a much wider impedance bandwidth with a compact size compared to Ref. [[30], [32]]. Furthermore, the terahertz antennas reported in Refs. [[14], [15], [16], [17], [18], [19], [20], [21], [22], [23], [24], [25], [26], [27], [28], [29], [30], [31], [32], [33], [34], [35]] do not possess the capability to radiate with circular polarization. In this regard, a few terahertz antenna designs with CP characteristics are listed in Refs. [[44], [45], [46], [47], [48], [49]]. In comparison to the referred THz CP antennas, the proposed design holds the most miniaturized size (0.83 $\lambda_{0}\times$ 0.43$\lambda_{0}$) and also shows the highest gain value of 8.65 dB along with the broadest 3 dB axial ratio bandwidth (ARBW). The broad ARBW confirms better circular polarization performance over the maximum range of frequencies.

**Table 2:** Summary of comparative analysis with previously reported references.

Ref.	Antenna Size $\lambda_{0}\times \lambda_{0}$	Operating Band (THz)	− 10 dB Bandwidth (THz)	3 dB ARBW (%)	Maximum Gain (dB)	Circular Polarization
[14]: 2022	2.57 × 1.85	0.52–0.78	0.26	NA	–	No
[15]: 2022	2.71 × 1.89	0.43–0.84	0.41	NA	–	No
[16]:2024	0.39 × 0.17	0.84–1.12	0.28	NA	7.41	No
[17]: 2022	0.98 × 0.49	0.276–0.711	0.435	NA	–	No
[18]: 2024	3.59 × 1.30	0.60–0.70	0.1	NA	–	No
[19]: 2022	3.28 × 2.10	0.5–0.8	0.3	NA	–	No
[20]: 2021	0.518 × 0.44	0.73–0.75	0.02	NA	6.395	No
[21]: 2023	0.96 × 0.67	3.572–3.6818	0.1098	NA	4.3	No
[22]: 2023	0.46 × 0.46	0.6–0.8	0.2	NA	7.62	No
[23]: 2023	0.34 × 0.26	0.57–1.02	0.45	NA	–	
[24]: 2023	0.61 × 0.61	0.445–0.470	0.025	NA	4.6	No
[25]: 2024	–	0.761–0.853 1.117–1.413	0.092 0.296	NA	7.31	No
[26]: 2022	–	1.281–1.354	0.073	NA	7.72	
[27]: 2023	–	1.267/1.502	–	NA	5.232	No
[28]: 2023	1.38 × 1.38	0.257–0.370	0.113	NA	2.45	No
[29]: 2021	0.5 × 0.5	0.095–0.205	0.11	NA	4.4	No
[30]: 2021	1.58 × 1.4	0.616–0.64	0.024	NA	9.45	No
[31]: 2023	0.27 × 0.18	1.41–3.0	1.59	NA	4.60	No
[32]: 2022	0.33 × 0.5	1.0	–	NA	8.87	No
[33]: 2022	0.71 × 0.35	0.35–0.75	0.4	NA	5.49	No
[34]: 2021	0.55 × 0.55	0.445–0.714	0.269	NA	5.7	No
[35]: 2023	0.44 ×0.44	0.3627–0.5918	0.2291	NA	4.0	No
[44]: 2020	1.2 ×0. 46	0.258–0.355	0.097	9.3 %	5.6	Yes
[45]: 2022	–	3.3688–3.7232	0.3544	2 %	8.0	Yes
[46]: 2020	1.1 × 0.55	0.98–1.225	0.245	8.8 %	2.59	Yes
[47]: 2021	1.1 × 0.55	0.98–1.23	0.25	8.7 %	2.61	Yes
[48]: 2022	–	0.5–0.7	0.2	–	5	Yes
[49]: 2024	–	2.31–2.41 3.11–3.51	0.1 0.4	–	6.11	Yes
**Proposed**	0.83 × 0.43	2.345–2.555	0.21	9.4 %	8.65	Yes (RHCP)

NA= Not Applicable.

In summary, the proposed array configuration is advantageous in terms of balanced performance by offering a smaller size, high gain, broad operating bandwidth, and right-hand circular polarization (RHCP) with a wider 3 dB AR bandwidth. Hence, the suggested array antenna could be promising for high-speed data transmission, material characterization, spectroscopy, and medical imaging applications in the terahertz regime due to its attractive size and superior performance.

## 7 Conclusion

In this article, an effort has been made to design a new 1 × 2 array antenna with a circular polarization using a single feed technique. The proposed graphene array antenna is intended for terahertz band applications. The intended geometry of the single antenna element has been obtained through the execution of six development stages. The design methodology includes the connection of a folded feed line, corner truncation at the patch, and the insertion of tiny flower-shaped slots placed in each quadrant of the patch along with the presence of a circular-shaped slot. The modified single-element patch antenna shows transformed polarization from linear to circular with enhanced 3 dB AR bandwidth performance. Finally, a 1× 2 array is configured comprising of two single-element patches, which are fed together by a magic-T power divider. Furthermore, the prescribed array antenna demonstrates an upgraded performance in terms of gain, impedance bandwidth, efficiency as well as the bandwidth for the axial ratio (3 dB) and the reflection coefficient. The suggested circularly polarized terahertz array antenna measures a compact geometry with an overall size of 102 × 53.5× 1.56 on a micrometer scale. Furthermore, it offers IBW of 210 GHz (2.345–2.555 THz), 3 dB ARBW of 205 GHz (2.345–2.55 THz), maximum gain, of 8.65 dB, and radiation efficiency of 99.8 %, respectively. This suggested circularly polarized microstrip 1 × 2 array antenna configuration is dedicated to work for wireless communications in the Terahertz (THz) spectral band precisely at around 2.45 THz. Therefore, it can be utilized for high-speed data transmission, material characterization, spectroscopy, and medical imaging applications in the terahertz domain.

## Funding

This research was funded by 10.13039/501100004242Princess Nourah bint Abdulrahman University Researchers Supporting Project number (PNURSP2024R51), Princess Nourah bint Abdulrahman University, Riyadh, Saudi Arabia.

## Data availability statement

The datasets are available within the manuscript.

## CRediT authorship contribution statement

**Abdelaaziz El Ansari:** Writing – original draft, Software, Investigation, Formal analysis, Data curation, Conceptualization. **Sudipta Das:** Writing – original draft, Validation, Supervision, Software, Investigation, Conceptualization. **Tanvir Islam:** Writing – review & editing, Visualization, Software, Formal analysis, Conceptualization. **Varakumari Samudrala:** Writing – review & editing, Visualization, Validation, Data curation, Conceptualization. **Naglaa F. Soliman:** Writing – review & editing, Methodology, Funding acquisition, Formal analysis, Data curation. **Abeer D. Algarni:** Writing – review & editing, Project administration, Methodology, Funding acquisition, Formal analysis. **Najiba El Amrani El Idrissi:** Writing – review & editing, Validation, Supervision, Resources, Methodology.

## Declaration of competing interest

The authors declare that they have no known competing financial interests or personal relationships that could have appeared to influence the work reported in this paper.
